# Female gender lost protective effect against disease progression in elderly patients with chronic hepatitis B

**DOI:** 10.1038/srep37498

**Published:** 2016-11-28

**Authors:** Hong You, Yuanyuan Kong, Jinlin Hou, Lai Wei, Yuexin Zhang, Junqi Niu, Tao Han, Xiaojuan Ou, Xiaoguang Dou, Jia Shang, Hong Tang, Qing Xie, Huiguo Ding, Hong Ren, Xiaoyuan Xu, Wen Xie, Xiaoqing Liu, Youqing Xu, Yujie Li, Jie Li, Shein-Chung Chow, Hui Zhuang, Jidong Jia

**Affiliations:** 1 Liver Research Center, Beijing Friendship Hospital, Capital Medical University; Beijing Key Laboratory of Translational Medicine on Liver Cirrhosis; National Clinical Research Center for Digestive Disease, Beijing, 100050, China; 2 Nanfang Hospital, Southern Medical University, Guangzhou, 510515, China; 3 Peking University People’s Hospital, Beijing, 100044, China; 4 First Affiliated Hospital of Xinjiang Medical University, Urumqi, 830054, China; 5 First Hospital of Jilin University, Changchun, 130021, China; 6 Tianjin Third Central Hospital, Tianjin Medical University, Tianjin, 300170, China; 7 Shengjing Hospital of China Medical University, Shenyang, 110022, China; 8 Henan Provincial People’s Hospital, Zhengzhou, 450003, China; 9 West China Hospital of Sichuan University, Chengdu, 610041, China; 10 Shanghai Ruijin Hospital, Jiao Tong University School of Medicine, Shanghai, 200025, China; 11 Beijing Youan Hospital, Capital Medical University, Beijing, 100069, China; 12 The Second Affiliated Hospital of Chongqing Medical University, Chongqing, 400010, China; 13 Peking University First Hospital, Beijing, 100034, China; 14 Beijing Ditan Hospital, Capital Medical University, Beijing, 100015, China; 15 Peking Union Medical College Hospital, Beijing, 100730, China; 16 Beijing Tiantan Hospital, Capital Medical University, Beijing, 100050, China; 17 The Third People’s Hospital of Taiyuan, Taiyuan, 030012, China; 18 Peking University Health Science Center, Beijing, 100191, China; 19 Department of Biostatistics & Bioinformatics, Duke University School of Medicine, Durham, 27710, USA

## Abstract

Female gender and younger age are protective factors against disease progression in chronic hepatitis B (CHB). However, it is not clear whether the disease progression still remains slow in elderly females. This study investigated the interaction of female gender and older age on the development of cirrhosis in patients recorded in China Registry of Hepatitis B. A total of 17,809 CHB patients were enrolled in this multi-center cross-sectional study. The prevalence of cirrhosis in female CHB patients increased faster than that in male CHB patients over 50 years old. Multivariate analysis showed that the increase of adjusted ORs for developing cirrhosis in females started to accelerate after 50 years old: 11.19 (95% CI: 5.93–21.11) in women versus 14.75 (95% CI: 8.35–26.07) in men at ages of 50–59 years, 21.67 (95% CI: 11.05–42.47) versus 24.4 (95% CI: 13.00–45.80) at ages 60–69 years, and 18.78 (95% CI: 6.61–53.36) versus 12.09 (95% CI: 4.35–33.61) in those over 70 years. In conclusion, the protective effect of female gender against cirrhosis gradually lost with increasing age, therefore disease progression should be monitored more closely in elderly women with CHB.

Chronic infection of hepatitis B virus (HBV) is a worldwide public health problem and poses enormous disease burden in Asia and other endemic areas^1^. Although universal HBV vaccination program has led to a dramatic decline in the prevalence of HBsAg in general population of China (from 9.75% in 1992 to 7.18% in 2006), CHB is still the predominant cause of cirrhosis and hepatocellular carcinoma (HCC) in this country^2^.

The well-known REVEAL study demonstrated that HBeAg positivity and high viral load are key drivers for progression to cirrhosis and HCC^3^. In addition, host factors including male gender, older age, heavy alcohol intake and higher body mass index also pose higher risks for disease progression^4^. Therefore, major international guidelines recommend a more aggressive antiviral therapy and closer monitoring for disease progression in male patients over 40 years^5^,^6^,^7^. In recent years, studies on chronic hepatitis C (CHC) or nonalcoholic fatty liver disease (NAFLD) suggested that the progression of fibrosis is accelerated in elderly women^8^,^9^. However, it is unknown whether the disease progression still remains slow in elderly female patients with CHB.

Therefore, to determine interaction of female gender and older age in the development of HBV-related cirrhosis, we carried out a cross-sectional study on a large database retrieved from China Registry of Hepatitis B (CR-HepB), which is a hospital-based, multi-center registry initiated in June 30, 2012^10^.

## Results

### Demographics and clinical characteristics of patients

**Figure 1:**
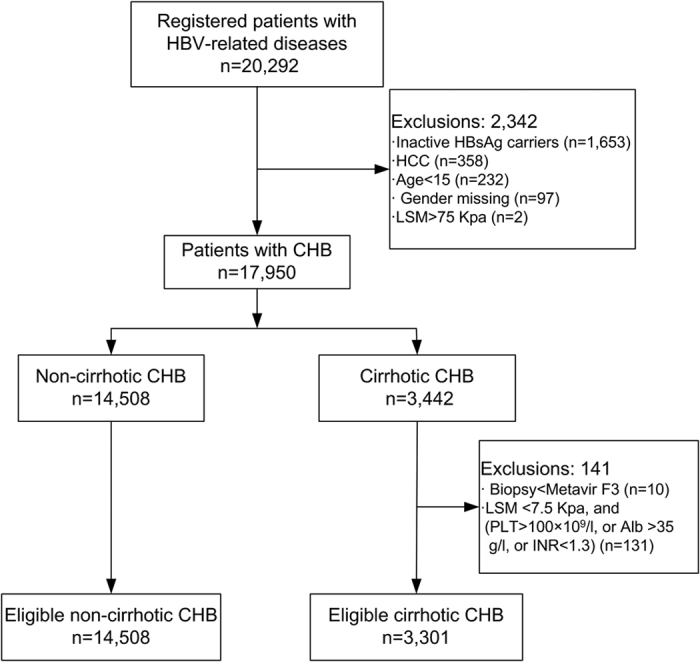
Flowchart of selection of patients with non-cirrhotic and cirrhotic CHB.

A total of 20,292 patients positive for HBsAg were registered in CR-HepB from June 30, 2012 to March 28, 2014. After excluding inactive HBsAg carriers, HCC and further logic checking, finally 17,809 patients including 14,508 cases of non-cirrhotic CHB and 3301 cases of cirrhotic CHB were analyzed (Fig. 1). Overall slightly more male patients received antiviral therapy than female patients (65.5% vs. 54.8%, *P* < 0.01). However, similar proportions of male and female patients received antiviral therapy in those older than 50 years (54.3% vs. 53.3%, *P* = 0.44). Demographic and clinical characteristics of the study population are listed in Table 1.

### The age- and sex- distributions in patients with non-cirrhotic CHB and cirrhotic CHB

In patients with non-cirrhotic CHB, majority of male and female patients were in the age group of 30–39 years, accounting for 30.6% and 26.2% of male and female patients, respectively. In patients with cirrhotic CHB, male patients at the age groups of 40–49 years and 50–59 years accounted for 30.8% and 31.8% of the total male cases, respectively; whereas female patients at the age group 50–59 years and 60–69 years accounted for 34.5% and 26.3% of the total female cases, respectively (Table 2). This indicates that the age of female cirrhotic patients was much older than male cirrhotic patients (*P* < 0.001).

**Figure 2:**
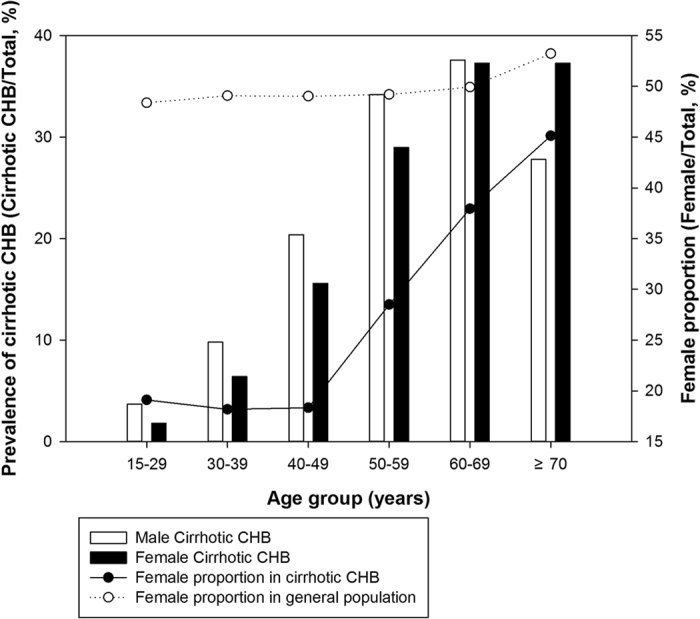
The age- and sex-specific prevalence of cirrhotic CHB and female proportion in cirrhotic CHB and general population. A stepwise increase was found in age-specific prevalence of cirrhotic CHB in females. The trend of female proportion in patients with cirrhotic CHB increased significantly faster than that in the general population (*P* = 0.024).

Although male CHB patients had high overall prevalence of cirrhosis than female patients (prevalence difference, −1.63%; Table 2), there seemed to be a turning point in age-specific prevalence. Under 59 years, the prevalence of cirrhosis was significantly lower in female patients than in male patients, and the differences increased with increasing age groups; at ages of 60–69 years it was similar to that in male patients, and after 70 years it was significantly higher than that in males (Fig. 2, Table 2).

Furthermore, trend analysis demonstrated that the proportion of females in patients with cirrhotic CHB increased significantly faster than that in the general population after 50 years (*F* = 40.66, *P* = 0.024). The highest female proportion was found in cirrhotic patients aged ≥70 years (45.13%, 95% CI: 38.14–52.11%) (Fig. 2, Supplementary Table 1).

### The impacts of sex difference and age-sex interaction on the risk for cirrhosis

**Figure 3:**
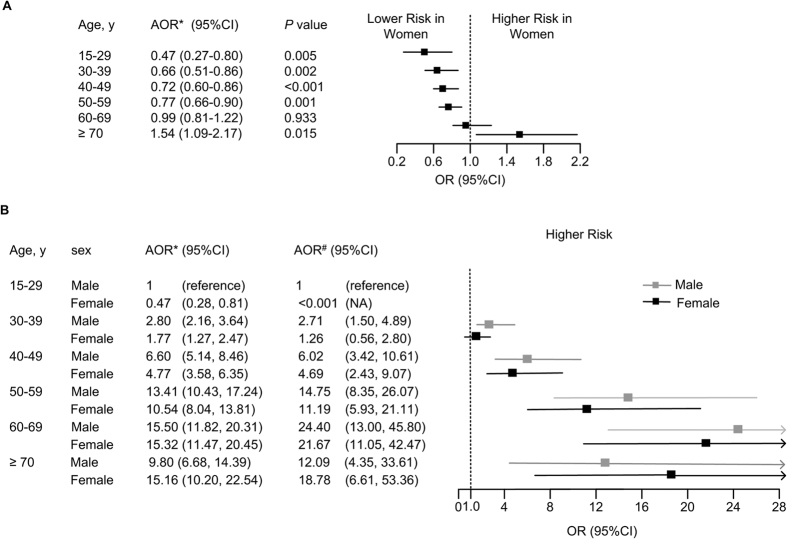
The impacts of gender-associated risk stratified by age (**A**) and age-sex interaction (**B**) on the development of cirrhosis in multivariate logistic regression models. Multivariate analyses indicated that the risk for cirrhosis started to accelerate at the age of 50 years in women, gradually approached the risk of men at the ages 60–69, and eventually overtook that in men after 70 years. NA: not available. ^*^ORs were adjusted for sex ratio in general population. ^#^ORs were adjusted for sex ratio in general population, HBV DNA level, HBeAg status, and levels of AST and ALT.

In patients before age of 50 years, the women were associated with a much lower risk for cirrhosis than the men (ORs: 0.47–0.72, 95% CI: 0.27–0.86). However, in those older than 50 years the risk for cirrhosis in women increased in a stepwise manner: slightly increased in the age group of 50–59 years (OR: 0.77, 95% CI: 0.66–0.90), approached the level of men in the age group of 60–69 years (OR: 0.99, 95% CI: 0.81–1.22), and finally overtook the men older than 70 years (OR: 1.54, 95% CI: 1.09–2.17) (Fig. 3A).

Similar results of sex-age interaction were derived from 2 types of multivariate logistic regression models (Fig. 3B). The risk of developing cirrhosis steadily increased with ages in both sexes. However, multivariate analysis showed that the increase of adjusted ORs for developing cirrhosis in females started to accelerate after 50 years: the adjusted ORs increased from 11.19 (95% CI: 5.93–21.11) at ages 50–59 years to 21.67 (95% CI: 11.05–42.47) at ages 60–69 years in females, with an increase rate of 93.7%; whereas in males the ORs increased from 14.75 (95% CI: 8.35–26.07) to 24.4 (95% CI: 13.00–45.80). with an increase rate of 65.4%. Furthermore, the risk of cirrhosis for women eventually overtook that in men over 70 years [18.78 (95% CI: 6.61–53.36) vs. 12.09 (95% CI: 4.35–33.61)].

### Faster progression of liver fibrosis among elderly female patients

**Figure 4:**
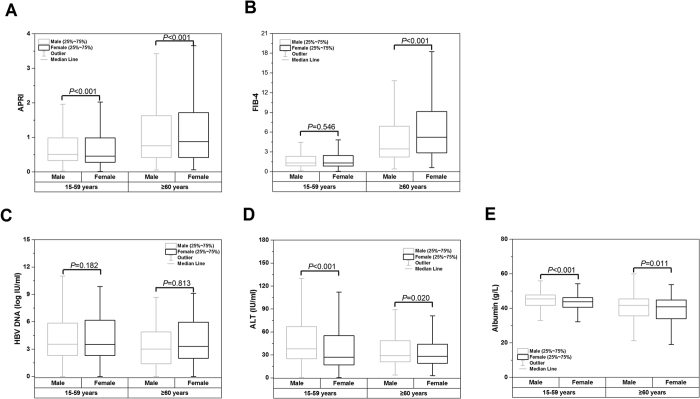
Comparisons of fibrosis stage evaluated by APRI (**A**) and FIB-4 (**B**), and levels of HBV DNA (**C**), ALT (**D**), and albumin (**E**) between male and female patients stratified by age group. The fibrosis stage was higher in female patients aged ≥60 years, however, the gender disparity in viral and biochemical variables remained stable between the age groups 15–59 years and ≥60 years.

In comparison with age-matched male patients, the fibrosis stage evaluated by APRI and FIB-4 was lower among female patients aged 15–59 years, whereas it significantly higher among female patients aged ≥60 years (Fig. 4A,B). In contrast, gender differences in viral and biochemical variables including serum levels of HBV DNA, ALT, and albumin remained the same among the male and female patients, with a similar or lower level in female patients compared to male patients in both age groups (Fig. 4C–E). Detailed results on the trend of disease progression derived from linear regression model were presented in Supplementary Fig. 1 and Supplementary Table 2.

## Discussion

In this multi-center cross-sectional study, we demonstrated that the protective effect of female gender observed in patients with chronic HBV infection aged 15–49 years gradually lost in patients older than 50 years, with disease progression ultimately approaching or even overtaking the age-matched men over 60 years old.

This novel finding suggests that there is an interaction between older age and female gender in HBV-related liver disease progression. The major concern is the impact of competing cause of death on the interpretation of the study finding, especially for male CHB patients who are older than 60 years. Multivariate analysis provides further evidences to support our hypothesis: risk for developing cirrhosis increased rapidly in elderly females even after adjusted for the survival rate of sex ratio in general population, and other well-known risk factors such as virological and biochemical profiles^3^,^4^.

Furthermore, the prevalence of HBsAg in women found in national HBV survey^2^ or in volunteer blood donors^11^ actually was lower than that in men, so it is unlikely that the increased female proportion in cirrhosis after 50 years old is caused by gender disparity in prevalence of HBV infection.

In line with the above findings, noninvasive measurements showed significantly ascending trends of APRI and FIB-4, indicating that fibrosis progression accelerated in elderly females. In contrast, serum levels of HBV DNA, ALT, and albumin showed stable or slightly downward trends in both sexes, and the gender differences remained unchanged in both age groups, implying that the faster disease progression in elderly female patients is not due to higher viral load or more severe hepatic necroinflammation.

The underlying mechanism(s) of gender disparity in fibrosis progression are still unclear. Some studies demonstrated that sex hormone may be involved in HBV life cycle, the immune response to HBV infection, and the progression of associated liver diseases^12^. Previous reports showed that postmenopausal women with CHC or NAFLD had severer hepatic steatosis and faster progression of liver fibrosis^8^,^9^, whereas delayed menopause, taking oral contraceptives or postmenopausal hormone replacement therapy were associated with slower progression of liver fibrosis^13^,^14^. In this line, the lost of protective effect against progression of liver fibrosis in women older than 50 years could be explained by the decrease in estrogen level. This notion is supported by the average age of 50 at menopause in both southern and northern parts of China^15^,^16^.

The present study has several limitations. First, the registry might have patient selection bias. But the age and sex distribution of patients in our study is similar to that of a multi-center study of cirrhosis in Southern China^17^. Second, the cross-sectional study was not an optimal design to prove causality between gender/age and the development of HBV-related cirrhosis. Potential biases and confounders included the different participation rate or accessibility to medical care, and gender differences in severity or survival rate of cirrhosis and competing risk. Third, the diagnosis of cirrhosis was based on histology in only about 18% of the cases. Considering the main focus of our study is gender disparity in the development of cirrhosis and progression trend of fibrosis rather than concrete fibrosis staging, the noninvasive modalities would offer an acceptable accuracy in such a big number of patients. Fourth, the registry did not collect data on HBV genotype, smoking, alcohol intake, or use of hormone replacement therapy. But this is unlikely to cause an increased risk for developing cirrhosis in females since the rates of smoking and drinking were much lower in Chinese women than that in men^18^,^19^ and the HBV genotype have no significant gender disparity^20^. In addition, the percentage of hormone replacement therapy in post-monopause women was only 2.1% in China women^21^. These few patients may not lead to a pivotal effect of the conclusion.

In conclusion, this multi-center cross-sectional study demonstrated that the protective effect of female gender against the development of HBV-related cirrhosis gradually lost after the age of 50 years. This meaningful finding would justify closer monitoring for disease progression in older women with chronic HBV infection.

## Methods

### Data sources and patients

We used the CR-HepB data from the initial 18 hospitals located in different areas of China from June 30, 2012 to March 28, 2014. Outpatients were recruited to this registry if they had been HBsAg positive for more than 6 months. The key clinical and laboratory data of patients were collected.

The enrollment criteria for this cross-sectional study were patients diagnosed as CHB with or without cirrhosis and with the following baseline information available: demography (age and sex), hematology (counts of white blood cells and platelets), biochemistry (alanine transaminase, ALT; aspartate transaminase, AST; total bilirubin, TB; albumin, Alb; and alpha-fetoprotein, AFP), and virology (hepatitis B surface antigen, HBsAg; hepatitis B e antigen, HBeAg; anti-hepatitis B e, anti-HBe; and HBV DNA).

The Ethics Committee of Beijing Friendship Hospital, Capital Medical University approved the study protocol (BJFH 2014033).

### Diagnosis of different phases of CHB infection

Inactive HBsAg carriers was defined as those with HBsAg positive for more than 6 months but with low HBV DNA and persistently normal ALT and without clinical, hematological, biochemical and radiological/endoscopic evidence of CHB or cirrhosis^5^,^6^,^7^.

CHB was diagnosed in the presence of elevated serum levels of ALT and HBV DNA ≥2000 IU/mL among those with HBsAg positive for more 6 months^5^,^6^,^7^.

Compensated cirrhosis was diagnosed based on liver biopsy (Ishak fibrosis stage 5–6 or METAVIR F4, evaluated by pathologists in each hospital)^22^ or a combination of unequivocal clinical and compatible laboratory manifestations (*e.g*., thrombocytopenia, leucopenia, hypoalbuminemia, hyperbilirubinemia and prolonged prothrombin time), radiology (*e.g*., irregular surface, coarsened texture or nodularity of the liver, enlargement of the left lobe, and splenomegaly)/transient elastography (LSM >13 kpa), and endoscopic findings (*e.g*., gastroesophageal varices)^5^,^6^,^7^.

Decompensated cirrhosis was diagnosed based on the presence of ascites confirmed by ultrasound, bleeding esophageal varices and/or hepatic encephalopathy in cirrhotic patients^4^,^23^.

### Evaluation of Liver fibrosis

Liver fibrosis was evaluated by noninvasive methods including AST-platelet index (APRI), and a fibrosis index based on four factors (FIB-4)^24^.

### Statistical analysis

Descriptive statistics including median, lower quartile, and upper quartile were used to describe the demographics and clinical characteristics of the patients.

The age- and sex- distribution in patients with non-cirrhotic and cirrhotic CHB were calculated. The prevalence of cirrhotic CHB was calculated by dividing the number of cirrhotic CHB by the total number of CHB in each sex and stratified by age group. The prevalence differences of cirrhosis between female and male patients in each age group and their 95% confidence intervals (CIs) were also obtained. The differences were considered significant if their 95% CIs did not include 0.

The proportions of female patients in different age groups were calculated to estimate the gender variations in patients with cirrhotic CHB. Furthermore, the trend of female proportion in cirrhosis was compared with that in the general population of China^25^ by using linear regression model.

Considering the unbalanced life expectancy, odds ratios (ORs) were adjusted for the sex ratio in general population^24^ to address the gender-associated risk of cirrhosis at each age group by using multivariate logistic regression model. The impact of age-sex interaction on the development of cirrhosis was analyzed with multivariate logistic regression models by using male patients aged 15–29 years as the reference group. Two predefined multivariable models were applied: a basic model adjusted only for sex ratio in general population^25^, and a full model further adjusted for HBV DNA level (≤5.0 log IU/mL or >5.0 log IU/mL), HBeAg status (negative or positive), and levels of AST (≤40 IU/ml or >40 IU/ml) and ALT (≤40 IU/mL or >40 IU/mL). The adjusted ORs were considered significantly different if the 95% CIs did not contain 1.

APRI, FIB-4, and levels of HBV DNA, ALT and Alb were used to evaluate the progression of liver disease among male and female patients stratified by two age groups: 15–59 years and ≥60 years. *P* value below 0.05 was considered statistically significant.

## Additional Information

**How to cite this article**: You, H. *et al*. Female gender lost protective effect against disease progression in elderly patients with chronic hepatitis B. *Sci. Rep.* **6**, 37498; doi: 10.1038/srep37498 (2016).

**Publisher’s note:** Springer Nature remains neutral with regard to jurisdictional claims in published maps and institutional affiliations.

## Supplementary Material

Supplementary Information

## Figures and Tables

**Table 1:** Characteristics of the registered CHB patients.

	Non-cirrhotic CHB			Cirrhotic CHB		
	Total n = 14,508	Male n = 10,290	Female n = 4218	Total n = 3301	Male n = 2414	Female n = 887
Age, year	40 (32, 50)	40 (32, 48)	40 (31, 52)	51 (43, 59)	50 (42, 58)	56 (48, 62)
HBeAg positive rate, %	3761 (56.8%)	2684 (55.9%)	1077 (59.2%)	494 (44.4%)	363 (41.8%)	131 (53.5%)
HBV DNA, log IU/ml	3.64 (2.30, 6.05)	3.66 (2.30, 5.94)	3.60 (2.30, 6.24)	2.58 (0, 4.79)	2.63 (0, 4.84)	2.56 (0, 4.49)
PLT count, ×10^9^/L	172.0 (133.0, 210.0)	169.0 (131.0, 208.0)	179.0 (136.0, 220.0)	94.0 (59.0, 139.0)	97.0 (63.0, 143.0)	84.0 (52.1, 133.0)
ALT, IU/mL	35.0 (22.0, 66.1)	38.0 (24.0, 70.0)	27.0 (17.0, 55.4)	32.1 (23.0, 49.0)	34.0 (24.0, 49.8)	28.9 (19.9, 45.0)
AST, IU/mL	30.0 (22.5, 48.7)	30.3 (23.0, 49.0)	28.0 (20.7, 47.0)	32.6 (22.0, 51.0)	33.0 (23.0, 50.0)	32.0 (20.0, 52.0)
Albumin, g/L	45.1 (42.0, 47.4)	45.6 (42.4, 47.8)	44.0 (41.2, 46.3)	40.7 (34.0, 45.6)	41.4 (34.5, 46.0)	38.6 (32.9, 44.0)
Bilirubin, 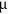 mol/L	13.8 (10.5, 18.5)	14.6 (11.2, 19.5)	12.1 (9.2, 15.4)	19.0 (13.2, 28.7)	19.2 (13.5, 29.2)	17.8 (12.6, 26.5)
CHE, IU/L	8058.0 (6633.5, 9590.5)	8426.5 (6788.0, 9891.0)	7595.0 (6222.0, 8692.0)	4868.5 (3039.5, 7108.0)	4803.0 (3043.0, 7277.0)	4877.0 (2973.0, 6615.5)
ALP, U/L	72.0 (59.0, 91.0)	75.0 (61.0, 92.0)	66.0 (54.0, 86.0)	86.0 (67.0, 117.0)	86.0 (68.0, 117.0)	87.0 (64.1, 117.0)
GGT, U/L	26.0 (17.0, 47.3)	30.0 (20.0, 55.1)	18.0 (13.0, 31.2)	45.0 (25.3, 80.0)	49.0 (28.0, 87.0)	36.0 (22.0, 63.0)
Cr, 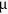 mol/L	71.6 (61.0, 81.5)	75.0 (67.0, 84.0)	55.0 (49.0, 63.7)	69.0 (58.0, 80.0)	72.3 (64.0, 83.0)	55.3 (48.0, 67.0)
Cholesterol, mmol/L	4.3 (3.8, 5.1)	4.3 (3.7, 5.1)	4.5 (3.9, 5.1)	3.6 (2.9, 4.4)	3.7 (3.0, 4.5)	3.6 (2.9, 4.3)
Triglyceride, mmol/L	1.1 (0.8, 1.7)	1.2 (0.8, 1.8)	1.0 (0.7, 1.4)	0.9 (0.6, 1.3)	0.9 (0.7, 1.3)	0.8 (0.6, 1.1)
PTA, %	102.0 (88.0, 118.0)	101.0 (87.0, 117.0)	103.0 (90.0, 120.0)	74.0 (59.0, 90.0)	72.0 (58.0, 89.0)	76.0 (63.3, 92.0)
INR, ratio	1.0 (0.9, 1.1)	1.0 (0.9, 1.1)	1.0 (0.9, 1.0)	1.1 (1.0, 1.3)	1.2 (1.0, 1.3)	1.1 (1.0, 1.3)

Data were presented by median (lower quartile, upper quartile).

CHB, chronic hepatitis B; PLT, platelet; ALT, alanine aminotransferase; AST, aspartate aminotransferase; CHE, cholinesterase; ALP, alkaline phosphatase; GGT, gamma glutamyltransferase; Cr, creatinine; PTA, prothrombin activity; INR, international normalized ratio of prothrombin time.

**Table 2:** Age- and sex- distributions in patients with CHB.

Age group (years)	Non-cirrhotic CHB n (%)			Cirrhotic CHB n (%)			Prevalence of cirrhosis^#^(%)		Prevalence Difference^†^ (95% CI)
	**Total**	**Female**	**Male**	**Total**	**Female**	**Male**	**In female**	**In male**	
15–29	2778 (19.1)	923 (21.9)	1855 (18.0)	89 (2.7)	17 (1.9)	72 (3.0)	1.81	3.73	−1.93 (−2.78, −1.08)
30–39	4249 (29.3)	1105 (26.2)	3144 (30.6)	418 (12.7)	76 (8.6)	342 (14.2)	6.44	9.81	−3.38 (−4.68, −2.07)
40–49	3809 (26.3)	903 (21.4)	2906 (28.2)	911 (27.6)	167 (18.8)	744 (30.8)	15.61	20.38	−4.78 (−6.79, −2.77)
50–59	2223 (15.3)	748 (17.7)	1475 (14.3)	1074 (32.5)	306 (34.5)	768 (31.8)	29.03	34.24	−5.21 (−7.73, −2.69)
60–69	1023 (7.1)	391 (9.3)	632 (6.1)	614 (18.6)	233 (26.3)	381 (15.8)	37.34	37.61	−0.27 (−3.78, 3.24)
≥70	426 (2.9)	148 (3.5)	278 (2.7)	195 (5.9)	88 (9.9)	107 (4.4)	37.29	27.79	9.50 (3.91, 15.08)
Total	14,508 (100%)	4218 (100%)	10,290 (100%)	3301 (100%)	887 (100%)	2414 (100%)	17.38	19.00	−1.63 (−2.58, −0.68)

^†^Prevalence Difference, prevalence of cirrhosis in females minus that in males within each age group; CI, confidence interval.

^#^Prevalence of cirrhosis was calculated by dividing the number of cirrhotic CHB by total number of CHB stratified by sex in each age group.

## References

[b1] Schweitzer A., Horn J., Mikolajczyk R. T., Krause G. & Ott J. J. Estimations of worldwide prevalence of chronic hepatitis B virus infection: a systematic review of data published between 1965 and 2013. Lancet 386, 1546–1555 (2015). 26231459 10.1016/S0140-6736(15)61412-X

[b2] Liang X. . Epidemiological serosurvey of hepatitis B in China–declining HBV prevalence due to hepatitis B vaccination. Vaccine 27, 6550–6557 (2009). 19729084 10.1016/j.vaccine.2009.08.048

[b3] Iloeje U. H., Yang H. I. & Chen C. J. Natural history of chronic hepatitis B: what exactly has REVEAL revealed? Liver Int. 32, 1333–1341 (2012). 22510145 10.1111/j.1478-3231.2012.02805.x

[b4] Fattovich G., Bortolotti F. & Donato F. Natural history of chronic hepatitis B: Special emphasis on disease progression and prognostic factors. J Hepatol 48, 335–352 (2008). 18096267 10.1016/j.jhep.2007.11.011

[b5] European Association For The Study Of The Liver. EASL clinical practice guidelines: Management of chronic hepatitis B virus infection. J Hepatol 57, 167–185 (2012). 22436845 10.1016/j.jhep.2012.02.010

[b6] Sarin S. K. . Asian-Pacific clinical practice guidelines on the management of hepatitis B: a 2015 update. Hepatol Int 10, 1–98 (2016). 10.1007/s12072-015-9675-4 PMC4722087 26563120

[b7] Terrault N. A. . AASLD guidelines for treatment of chronic hepatitis B. Hepatology 63, 261–283 (2016). 26566064 10.1002/hep.28156 PMC5987259

[b8] Codes L. . Liver fibrosis in women with chronic hepatitis C: evidence for the negative role of the menopause and steatosis and the potential benefit of hormone replacement therapy. Gut 56, 390–395 (2007). 17005762 10.1136/gut.2006.101931 PMC1856786

[b9] Yang J. D. . Gender and menopause impact severity of fibrosis among patients with nonalcoholic steatohepatitis. Hepatology 59, 1406–1414 (2014). 24123276 10.1002/hep.26761 PMC3966932

[b10] Jia J. D. . CR-HepB: A new registry system for documenting and analyzing the demography, virology and medicine treatments of hepatitis B in China. Hepatology 58 (suppl), 619A (2013).

[b11] Yang Y. L. . Hepatitis B surface antigen variants in voluntary blood donors in Nanjing, China. Virol J 9, 82 (2012). 22500577 10.1186/1743-422X-9-82 PMC3342217

[b12] Wang S. H., Chen P. J. & Yeh S. H. Gender disparity in chronic hepatitis B: Mechanisms of sex hormones. J Gastroenterol Hepatol 30, 1237–1245 (2015). 25708186 10.1111/jgh.12934

[b13] Yu M. W. . Role of reproductive factors in hepatocellular carcinoma: impact on hepatitis B- and C-related risk. Hepatology 38, 1393–1400 (2003). 14647050 10.1016/j.hep.2003.09.041

[b14] Xi B., He D., Hu Y. & Zhou D. Prevalence of metabolic syndrome and its influencing factors among the Chinese adults: the China Health and Nutrition Survey in 2009. Prev Med. 57, 867–871 (2013). 24103567 10.1016/j.ypmed.2013.09.023 PMC4044099

[b15] Li L. . Factors associated with the age of natural menopause and menopausal symptoms in Chinese women. Maturitas 73, 354–360 (2012). 23026018 10.1016/j.maturitas.2012.09.008

[b16] Liu K. . Relationship between menopause and health-related quality of life in middle-aged Chinese women: a cross-sectional study. BMC Womens Health 14, 7 (2014). 24410885 10.1186/1472-6874-14-7 PMC3893455

[b17] Wang X. . Study of liver cirrhosis over ten consecutive years in Southern China. World J Gastroenterol 20, 13546–13555 (2014). 25309085 10.3748/wjg.v20.i37.13546 PMC4188906

[b18] Millwood I. Y. . Alcohol consumption in 0.5 million people from 10 diverse regions of China: prevalence, patterns and socio-demographic and health-related correlates. Int J Epidemiol 42, 816–827 (2013). 23918852 10.1093/ije/dyt078 PMC3733702

[b19] Lv J. . Gender-specific association between tobacco smoking and central obesity among 0.5 million Chinese people: the China Kadoorie Biobank Study. PLoS One 10, e0124586 (2015). 25897789 10.1371/journal.pone.0124586 PMC4405570

[b20] Liu C. J. & Kao J. H. Global perspective on the natural history of chronic hepatitis B: role of hepatitis B virus genotypes A to J. *Semin Liver Dis* 33, 97–102 (2013). 10.1055/s-0033-1345716 23749665

[b21] Jin F. . Knowledge and attitude towards menopause and hormone replacement therapy in Chinese women. Gynecol Obstet Invest 79, 40–45 (2015). 25277502 10.1159/000365172

[b22] Bedossa P. & Poynard T. An algorithm for the grading of activity in chronic hepatitis C. The METAVIR cooperative study group. Hepatology 24, 289–293 (1996). 8690394 10.1002/hep.510240201

[b23] Gines P., Quintero E. & Arroyo V. Compensated cirrhosis: natural history and prognosis. Hepatology 7, 122–128 (1987). 3804191 10.1002/hep.1840070124

[b24] European Association for Study of Liver; Asociacion Latinoamericana para el Estudio del Higado. EASLALEH Clinical Practice Guidelines: Noninvasive tests for evaluation of liver disease severity and prognosis. *J Hepatol* **63,** 237–264 (2015).

[b25] National Bureau of Statistics of the People’s Republic of China. China Statistical Yearbook, 1 ed. (Beijing: China Statistics Press, 2014).

