# Supplementary material for: Female gender lost protective effect against disease progression in elderly patients with chronic hepatitis B
Source: Sci Rep. 2016 Nov 28;6:37498. doi: 10.1038/srep37498 (PMC5124962; doi:10.1038/srep37498)
Supplement: Supplementary Information [file srep37498-s1.pdf]

Female gender lost protective effect against disease progression in elderly patients with chronic hepatitis B

Hong You[1 +], Yuanyuan Kong[1+], Jinlin Hou[2], Lai Wei[3], Yuexin Zhang[4], Junqi Niu[5], Tao Han[6], Xiaojuan Ou[1], Xiaoguang Dou[7], Jia Shang[8], Hong Tang[9], Qing Xie[10], Huiguo Ding[11], Hong Ren[12], Xiaoyuan Xu[13], Wen Xie[14], Xiaoqing Liu[15], Youqing Xu[16], Yujie Li[17], Jie Li[18], Shein-Chung Chow[19], Hui Zhuang[18], and Jidong Jia[1*]

[1] Liver Research Center, Beijing Friendship Hospital, Capital Medical University；Beijing Key Laboratory of Translational Medicine on Liver Cirrhosis；National Clinical Research Center for Digestive Disease, Beijing, 100050, China;

[2] Nanfang Hospital, Southern Medical University, Guangzhou, 510515, China;

[3] Peking University People's Hospital, Beijing, 100044, China;

[4] First Affiliated Hospital of Xinjiang Medical University, Urumqi, 830054, China;

[5] First Hospital of Jilin University, Changchun, 130021, China;

[6] Tianjin Third Central Hospital, Tianjin Medical University, Tianjin, 300170, China;

[7] Shengjing Hospital of China Medical University, Shenyang, 110022, China;

[8] Henan Provincial People's Hospital, Zhengzhou, 450003, China;

[9] West China Hospital of Sichuan University, Chengdu, 610041, China;

[10] Shanghai Ruijin Hospital, Jiao Tong University School of Medicine, Shanghai, 200025, China;

[11] Beijing Youan Hospital, Capital Medical University, Beijing, 100069, China;

[12] The Second Affiliated Hospital of Chongqing Medical University, Chongqing, 400010, China;

[13] Peking University First Hospital, Beijing, 100034, China;

[14] Beijing Ditan Hospital, Capital Medical University, Beijing, 100015, China;

[15] Peking Union Medical College Hospital, Beijing, 100730, China;

[16] Beijing Tiantan Hospital, Capital Medical University, Beijing, 100050, China;

[17] The Third People's Hospital of Taiyuan, Taiyuan, 030012, China;

[18] Peking University Health Science Center, Beijing, 100191, China;

[19] Department of Biostatistics & Bioinformatics, Duke University School of Medicine, Durham, 27710, USA.

* Corresponding：jia_jd@ccmu.edu.cn

[+] These authors contributed equally to this work

Supplementary Table 1 Proportions of female patients within cirrhotic CHB and general population stratified by age

Age group (years)	Cirrhotic CHB		General population† Female proportion (%)
	Female proportion (%)	(95% CI)	
15-29	19.10	(10.93, 27.27)	48.38
30-39	18.18	(14.48, 21.88)	49.08
40-49	18.33	(15.82, 20.84)	49.03
50-59	28.49	(25.79, 31.19)	49.20
60-69	37.95	(34.11, 41.79)	49.93
≥70	45.13	(38.14, 52.11)	53.22

† The age-specific female proportions in general population were cited from China Statistical Yearbook 2014 published by National Bureau of Statistics of the People's Republic of China.

CI, confidence interval.

Supplementary Table 2 Trends of fibrosis stage, viral and biochemical variables in elderly CHB patients stratified by sex

	Sex	Slope	95% CI	Equation
APRI	Female	0.02651 ± 0.01390	(-0.01771, 0.07073)	Y=0.02651*X+0.7154
	Male	-0.000694 ± 0.005348	(-0.01771, 0.01632)	Y=-0.000694*X+0.7926
FIB-4	Female	0.2252[†] ± 0.04955	(0.06754, 0.3829)	Y=0.2252*X+2.276
	Male	0.09976 ± 0.03586	(-0.01433, 0.2139)	Y=0.09976*X+2.452
HBV DNA, log IU/ml	Female	0.003112 ± 0.01794	(-0.05399, 0.06021)	Y=0.003112*X+2.897
	Male	0.009866 ± 0.02629	(-0.07379, 0.09352)	Y=0.009866*X+3.035
ALT, IU/mL	Female	-0.02900 ± 0.1351	(-0.4590, 0.4010)	Y=-0.02900*X+27.46
	Male	-0.4166 ± 0.1720	(-0.9639, 0.1307)	Y=-0.4166*X+34.90
Bilirubin, μmol/L	Female	-0.2040[†] ± 0.05008	(-0.3634, -0.04465)	Y=-0.2040*X+43.64
	Male	-0.1060[†] ± 0.01381	(-0.1499, -0.06206)	Y=-0.1060*X+43.17

[†] *P* value<0.05, the trend was considered statistically significant.

APRI, aspartate aminotransferase-platelet index; FIB-4, a fibrosis index based on four factors; ALT, alanine aminotransferase.

Supplementary Fig. 1

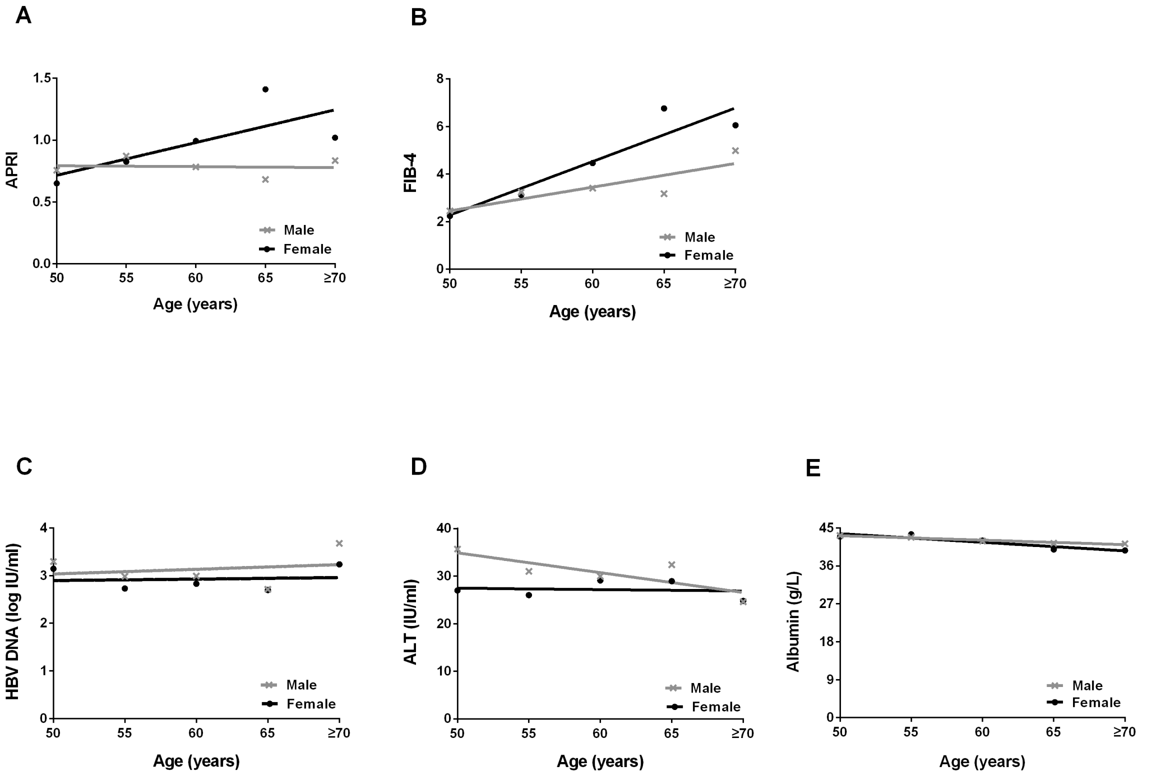

Trends of fibrosis stage evaluated by APRI (A) and FIB-4 (B), and serum levels of HBV DNA (C), ALT (D), and albumin (E). Trend analyses showed the ascending trends of the progression of fibrosis in elderly female patients, whereas the viral and biochemical variables remained stable or declined. See detailed results in Supplementary table 2.
